# Burden of fatigue in cryopyrin-associated periodic syndromes

**DOI:** 10.1016/j.ero.2025.03.003

**Published:** 2025-04-28

**Authors:** Özlem Satirer, Yilmaz Satirer, Anne-Kathrin Gellner, Susanne M. Benseler, Jasmin B. Kuemmerle-Deschner

**Affiliations:** 1 Paediatric Rheumatology, Department of Paediatrics and Autoinflammation Reference Center Tuebingen (arcT), University Hospital Tuebingen, Tuebingen, Germany; 2 European Reference Network – Rare Immunodeficiency, Autoinflammatory and Autoimmune Disease (ERN-RITA); 3 Department of Paediatrics, Zollernalb Klinikum, Balingen, Germany; 4 Department of Psychiatry and Psychotherapy, Bonn University Hospital, Bonn, Germany; 5 Rheumatology, Department of Paediatrics, Alberta Children's Hospital (ACH), ACH Research Institute, Cumming School of Medicine, University of Calgary, Calgary, AB, Canada; 6 Children's Health Ireland, Dublin, Ireland

## Abstract

**Objectives:**

Fatigue in cryopyrin-associated periodic syndromes (CAPS) often persists despite controlling inflammation. This study explores fatigue burden, its link to disease activity, and factors tied to chronic fatigue in autoinflammatory conditions.

**Methods:**

A single-centre study (2007-2024) of children and adults with CAPS analysed demographics, clinical data, inflammation, treatment, disease activity, and fatigue scores. Relationships between these factors and predictors of chronic fatigue were assessed.

**Results:**

A total of 108 patients with CAPS were included; 53 were female (49%). Median age at diagnosis was 11 years (range, 0-73); median follow-up duration 7 years (range, 1-18). Muckle-Wells syndrome was the most common phenotype (86%). At the time of diagnosis, hearing loss was present in 30%, aseptic meningitis in 6%. At enrolment, fatigue was the most common symptom, affecting 100 patients (93%), with a median fatigue score of 7 (0-10, visual analogue scale); high disease activity was present in 67%. At the last visit, fatigue remained prevalent, reported in 83 (77%), with a median score of 3 (range, 0-10). Importantly, only 4% of patients had high disease activity, whereas 83% had inactive disease. Factors influencing chronic fatigue in CAPS included age at diagnosis (*P* < .001, Cohen's *d* = −0.776, β = −2.15), NLRP3 mutation status (*P* = .009, Cohen's *d* = −0.516, β = −1.48), disease activity (*P* < .001), familial CAPS (62% vs 38%, *P* = .132, Cohen's *d* = −0.298, β = −0.87), and the COVID-19 pandemic (*P* < .001).

**Conclusions:**

Fatigue persists in autoinflammatory diseases despite effective disease control. Further research is needed to identify mechanisms and interventions.

**WHAT IS ALREADY KNOWN ON THIS TOPIC**

• Chronic fatigue is highly prevalent in children and adults with CAPS.

**WHAT THIS STUDY ADDS**

• While fatigue and disease activity correlated in CAPS, chronic fatigue persisted despite effective control of disease activity.
• High risk factors for chronic fatigue in CAPS were found to be age at diagnosis, NLRP3 variant, familial CAPS clustering, and the COVID-19 pandemic.

**HOW THIS STUDY MIGHT AFFECT RESEARCH, PRACTICE OR POLICY**

• The underlying mechanism for chronic fatigue remains unclear, highlighting the need for further research to identify targeted treatments.

Alt-text: Unlabelled box dummy alt text

## INTRODUCTION

Cryopyrin-associated periodic syndromes (CAPS) represent a spectrum of rare, hereditary autoinflammatory disorders, all of which are attributed to mutations in the *NLRP3* gene. This gene is responsible for encoding the cryopyrin protein, a critical component of the innate immune system. The conditions classified under CAPS include familial cold autoinflammatory syndrome (FCAS), Muckle-Wells syndrome (MWS), and chronic infantile neurological cutaneous and articular (CINCA) syndrome/neonatal-onset multisystem inflammatory disease (NOMID). These syndromes are collectively characterised by chronic systemic inflammation, which manifests in a variety of symptoms such as recurrent fevers, urticarial-like rash, arthralgia, and, most notably, profound fatigue [1].

Fatigue, within the context of CAPS, is recognised as one of the most debilitating and pervasive symptoms encountered by patients. Distinct from general tiredness, the fatigue experienced in CAPS is often severe, persistent, and significantly disproportionate to the level of physical activity or rest undertaken by the patient. This unrelenting fatigue substantially impairs daily functioning and drastically diminishes the overall quality of life [2].

Recent studies have demonstrated that interleukin-1 (IL-1) plays a central role in the inflammatory processes of CAPS. IL-1 is a key cytokine in the pathophysiology of these diseases, triggering the inflammatory cascade, leading to tissue damage and the development of various symptoms, particularly severe fatigue [3]. The existing literature reports that IL-1 inhibitors are effective in controlling the inflammatory manifestations of IL-1-related diseases, including CAPS, and in alleviating fatigue [4,5]. However, despite the control of disease activity through IL-1 pathway inhibition, fatigue often persists. Similarly, studies conducted in patients with familial Mediterranean fever (FMF) have shown that they experience significantly higher levels of fatigue compared with healthy individuals [3]. This persistent fatigue continues to negatively impact patients’ quality of life, leads to productivity loss, and imposes significant societal costs, similar to the economic burden of fatigue observed in long COVID [6].

Despite the prevalence of fatigue in such inflammatory conditions, the link between inflammation and fatigue remain inadequately understood. This gap in understanding is particularly evident in the context of CAPS, where the persistence of disabling fatigue, even after traditional inflammatory pathways have been addressed, continues to pose a significant challenge. The dramatic burden of fatigue urgently mandates more targeted research efforts aimed at characterising and quantifying fatigue in autoinflammation with the goal of developing more effective therapeutic interventions.

Therefore, the aims of the study were: (1) to describe a longitudinal cohort of consecutive paediatric and adult patients with CAPS focused on frequency and severity of fatigue; (2) to examine the trajectories of fatigue scores across prepandemic, pandemic, and postpandemic periods; and 3) to identify high-risk factors associated with the development of chronic fatigue in CAPS.

## METHODS

A single-centre study of consecutive paediatric and adult patients diagnosed with CAPS and followed between January 2007 and June 2024 was performed. Patients were included, if they (1) met the classification and/or diagnosis criteria for CAPS [7,8], (2) were ≥2 years of age at the baseline visit [9] and (3) were followed longitudinally at the centre for at least 12 months. Patients with comorbidities independently associated with fatigue including malignancies and defined mental health conditions were excluded. Patient data were prospectively captured in the designated electronic database, the institutional Arthritis and Rheumatism Database and Information System and retrospectively extracted for analysis in this study. Approval of the Ethics Committee of the Medical Faculty at the Eberhard Karls University and at the University Hospital Tuebingen was obtained (Project Number: 070/2024BO2).

### Data and assessments

Patient- and disease-related data included gender, ethnicity, age at symptom onset, age at diagnosis, and age at start of therapy. CAPS-associated clinical symptoms and the distinct CAPS phenotype (FCAS, MWS, and CINCA/NOMID) were captured. Laboratory parameters comprised inflammatory markers including serum amyloid A (SAA) and C-reactive protein (CRP) and *NLRP3* gene variants and their classification according to the American College of Medical Genetics and Genomics [10]. Treatments were documented including type, dose, and frequency of medication. Data were captured serially including at baseline and at all follow-up visits. These included (1) baseline visit defined as the initial assessment of patients conducted before start of therapy, (2) follow-up visits, subsequent serial evaluations performed after the start of therapy to monitor progress and response, and (3) the last follow-up visit, the concluding assessment of patients at the end of the study period after a minimum interval of 12 months.

### Definition of chronic fatigue

In adults, fatigue is defined as a persistent state of physical and/or mental exhaustion that is not alleviated by rest and significantly impairs daily functioning [11]. Similarly, in children, fatigue is characterised by a prolonged and debilitating lack of energy that impairs their ability to engage in age-appropriate activities and affects academic performance [12]. Chronic fatigue was defined according to the 2015 the Institute of Medicine criteria as fatigue lasting at least 6 months [12]. In this study, it was further specified as fatigue emerging at any time during follow-up and persisting for >6 months at the last visit.

### Monitoring instruments

Disease activity was captured using validated instruments, including the Physician Global Assessment (PGA) and the Patient/Parent Global Assessment (PPGA) [13]. Both instruments quantify disease activity along a 10 cm visual analogue scale (VAS), where 0 indicates no disease activity and 10 represents maximal disease activity. Categories of clinical disease activity were determined by PGA and PPGA on a 10 cm VAS and categorised as mild (<2), moderate (2-4), and high (>4) as described previously [14]. Fatigue was assessed using a 10 cm VAS within the standard PedsQL Present Functioning Visual Analogue Scales questionnaire [15], which has 6 parameters, including fatigue, and is routinely completed by patients before their outpatient visit. A score of 0 indicated no fatigue, whereas a score of 10 represented maximum fatigue. Fatigue was considered present if the VAS score was ≥1. The PedsQL VAS has been validated for both self-report by children (ages 5-18 years) and parent proxy-report (ages 2-18 years) [[15], [16], [17], [18]]. The number of school- or workdays missed over the past 3 months was considered a measure of impact of disease and was serially determined. For children ages below the age of 8 years and for individuals unable to self-report, information was provided by the caregiver. For children aged 8 to 12 years, the same report was collaboratively completed by both the parent and the child. For individuals age ≥12 years including adults, data were collected through self-reports. Interpreters facilitated communication for non-German-speaking patients, and written forms effectively addressed communication barriers for those with hearing impairments.

### Outcomes

Primary outcome was chronic fatigue defined as an elevated fatigue score ≥2 documented over at least 6 months. Secondary outcomes included: (1) fatigue scores over time, (2) fatigue scores 6 months before and 6 months after the COVID-19 pandemic's onset, (3) number of missed school- or workdays, and (4) disease activity scores and inflammatory markers over time.

### Putative risk factors for chronic fatigue in CAPS

Variables presumed to be associated with chronic fatigue were identified using a hypothesis driven approach based on published evidence and clinical observations. These included the patient's gender, age group at diagnosis of CAPS (childhood vs adults), *NLRP3* mutation classification (pathologic/likely pathologic vs variants of uncertain significance―VUS/none), physician derived disease activity estimate and familiar clustering of CAPS defined as at least one first degree related with CAPS.

### Analysis

Descriptive statistics summarised patient characteristics. Categorical variables were reported as frequencies, and continuous variables as means (SD) or medians (range), as appropriate. Missing data were handled using last observation carried forward. Normality was assessed with Shapiro-Wilk and Kolmogorov-Smirnov tests. Independent *t* tests were used for group comparisons. Longitudinal analyses employed repeated measures analysis of variance. Variables with *P* < .1 in univariate analyses were included in a multiple linear regression model to identify independent predictors of fatigue severity. Analyses were conducted using SPSS 28.0.1.1 (IBM, 2021).

## RESULTS

A total of 108 patients were included, comprising 55 males and 53 females. Four patients were excluded, 2 for malignancy, and 2 for being under 2 years of age. The median age of the cohort at disease onset was 3 years (range, 0-68) and at CAPS diagnosis 11 years (range, 0-73). Of the 108 patients, 69% were diagnosed in childhood, and 31% in adulthood. A total of 8% had the mild FCAS phenotype, 86% the moderate MWS, and 6% the severe CINCA/NOMID phenotype. *NLRP3* gene variants were (1) pathogenic/likely pathogenic variants in 42% including E311K and A439V, (2) VUS in 50% including Q703K and V198M, and (3) no variants in 8%. The most common *NLRP3* variants were Q703K (33%), A439V (15%), E311K (15%), and V198M (13%). A total of 90% were of German background, whereas the remainder were Turkish, Italian, and Albanian. All Turkish patients had the Q703K mutation. After diagnosis, 60% of patients were treated with canakinumab alone, 12% were started on anakinra of whom half were subsequently switched to canakinumab, and 28% were treated with colchicine only. The study included a total of 432 follow-up visits. The median follow-up time was 7 years (range, 1-18) (Table 1).

**Table 1:** Baseline characteristics of consecutive paediatric and adult patients with CAPS

Characteristics	CAPS cohort, n = 108
Gender, male:female	55:53
Age at disease onset, median (range), y	3 (0-68)
Age at diagnosis, median (range), y	11 (0-73)
Follow-up, median (range), y	7 (1-18)
CAPS phenotypes	
Mild phenotype (FCAS), n (%)	9 (8)
Moderate phenotype (MWS), n (%)	93 (86)
Severe phenotype (CINCA/NOMID), n (%)	6 (6)
Genotypes: *NLRP3* gene variants	
Pathogenic and likely pathogenic variants n (%)	45 (42)
Variant of uncertain significance, n (%)	54 (50)
No variant detected, n (%)	9 (8)
Age group at diagnosis	
Children, n (%)	74 (69)
Adults, n (%)	34 (31)
Initial treatment	
Canakinumab, n (%)	65 (60)
Colchicine, n (%)	30 (28)
Anakinra, n (%)	13 (12)

CAPS, cryopyrin-associated periodic syndromes; CINCA, chronic infantile neurological cutaneous and articular syndrome; FCAS, familial cold autoinflammatory syndrome; MWS, Muckle-Wells syndrome; n, number; NOMID, neonatal-onset multisystem inflammatory disease.

### Clinical features and inflammatory markers

At diagnosis, the most common CAPS symptoms were: (1) fatigue in 93% (100 patients), (2) arthralgia in 83% (90), and (3) myalgia in 56% (60). Fever attacks were noted in 55% (59), urticaria-like rash in 57% (61), and cold and stress-triggered attacks in 60% (64). Conjunctivitis affected 45% (49), whereas hearing loss and arthritis were observed in 30%, respectively. Raised inflammatory markers including CRP and/or SAA were detected in all patients at diagnosis. The median CRP level was 2.2 mg/dL (range, 0-17); the median SAA level was 14.3 mg/L (11-234).

### Disease activity

**Figure 1:**
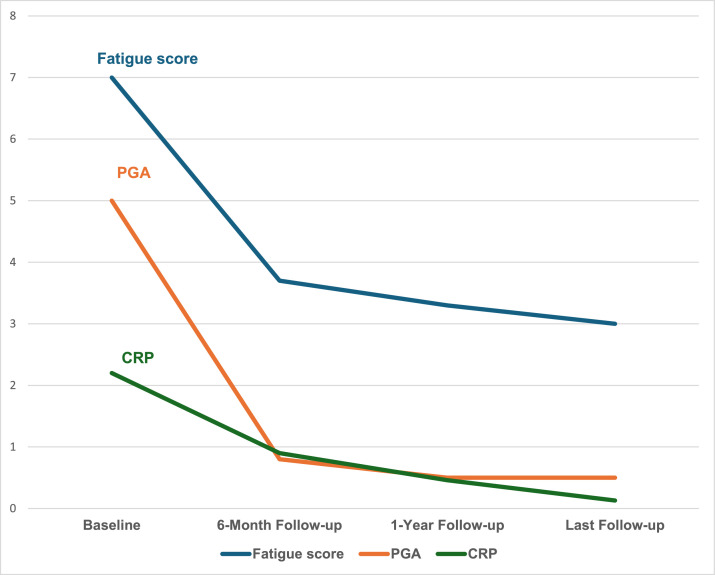
Trajectories of fatigue and disease activity over time in 108 patients with cryopyrin-associated periodic syndromes (CAPS). Median values for fatigue scores, PGA scores, and CRP levels at baseline, 6-month, 1-year, and last follow-up visits are shown. Fatigue and PGA scores were measured using a 10 cm visual analogue scale with higher scores reflecting greater severity. Fatigue scores declined from 7 (0-10) at baseline to 3 (0-10) at the last follow-up, whereas PGA scores improved from 5 (0-9) to 0.57 (0-8). CRP levels, with a clinical cutoff of 0.5 mg/dL, decreased from 2.2 mg/dL (0-17) at baseline to 0.13 mg/dL (0-5) at the last follow-up. Repeated measures analysis of variance (ANOVA) indicated significant differences across visits for fatigue score, PGA score, and CRP levels (*P* < .001). CRP, C-reactive protein (cutoff: 0.5 mg/dL); PGA, Physician Global Assessment.

At baseline, all patients had evidence of active disease with 67 patients (62%) having high disease activity, 35 patients (32%) moderate, and 6 (6%) mild disease activity. The median PGA score was 5 (range, 0-9), and the median PPGA score was 5 (range, 0-9). At the 6-months follow-up visit, only 40 patients remained symptomatic, whereas 68 patients (63%) had no evidence of disease activity. Mild disease activity was observed in 20 patients (19%), moderate in 17 (16%), and high disease activity in only 3 patients (2%). The median PGA score was 0.84 (range, 0-6), and the median PPGA score was 0.96 (range, 0-7). The median CRP level was 0.9 mg/dL (range, 0-3), and SAA 0.63 mg/L (range, 0-25). At the 12-months follow-up visit, 77 patients (71%) had no disease activity, whereas mild disease activity remained present in 21 (20%), moderate in 7 (7%), and high in 3 patients (2%). The median PGA score was 0.58 (range, 0-5), and the median PPGA 0.69 (range, 0-6). The median CRP level was 0.46 mg/dL (range, 0-4), and the median SAA 0.34 mg/L (range, 0-26). At the last follow-up visit, 83 patients (77%) had no disease activity, whereas 15 (14%) had mild, 6 (5%) moderate, and 4 (3%) had high disease activity. The median PGA score was 0.57 (range, 0-8), and the median PPGA 0.65 (range, 0-8). The median CRP level was 0.13 mg/dL (range, 0-5), and SAA 1.4 mg/L (range, 0-50). Trajectories are depicted in Figure 1.

### Fatigue

At baseline, 93% of patients reported fatigue, with a median fatigue score of 7 (range, 0-10). At the 6-months follow-up visit, fatigue was documented in 79%, median score decreased to 3.7 (range, 0-9). At the 12-months follow-up, 78% reported fatigue, with a median score of 3.3 (range, 0-10) and at last follow-up visit, 77% with a median score of 3 (range, 0-10). Disease activity and fatigue were significantly associated at all time points (baseline, *P* < .001; 6 months, *P* < .001; 12 months, *P* < .001, and last follow-up, *P* = .013) (Supplementary Table). Although both clinical and laboratory measures of disease activity normalised after start of therapy, fatigue scores remained elevated (see Fig 1).

### Impact of fatigue

Before the initiation of therapy, patients with active CAPS missed a median of 12.2 days of school or work over a 3-month period related to their disease (not including scheduled health care visits). After the start of therapy and effective control of disease activity, the median value decreased to 6.1 days over the same period. Data were available for 40 patients. Notably, at the last follow-up visit all 40 patients had no disease activity, however continued to experience fatigue; the median number of disease-related missed days from school or work over a 3-month period was 5.3 days, with a range of 1 to 11 days at their most recent visit.

### Trajectories of fatigue scores across prepandemic, pandemic, and postpandemic periods

**Figure 2:**
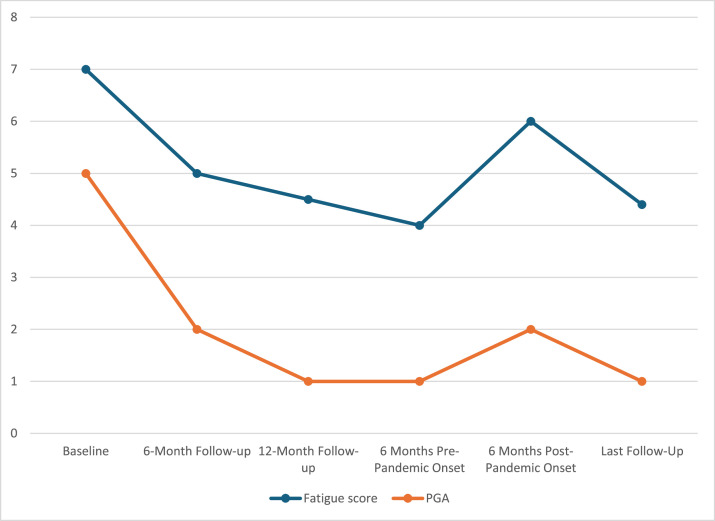
Trajectories of PGA and fatigue scores across prepandemic, pandemic, and postpandemic periods. Six months after therapy, the median fatigue score was 5 (range, 0-9), and the median PGA was 2 (range, 0-8). At 12 months, the median fatigue score remained 5 (range, 0-9), whereas the median PGA decreased to 1 (range, 0-5). Six months before the onset of the COVID-19 pandemic, the median fatigue score was 4 (range, 0-8), and the median PGA was 1 (range, 0-4). Six months after the onset, the median fatigue score increased to 6 (range 0-10), with a median PGA of 2 (range, 0-6). At the last follow-up, the median fatigue score was 4 (range, 0-9), and the median PGA remained 1 (range, 0-5). A repeated measures analysis of variance (ANOVA) revealed a statistically significant difference in both fatigue and PGA scores over time (*P* < .01). PGA, Physician Global Assessment.

The impact of the COVID-19 pandemic on fatigue was assessed by comparing scores from 6 months before and 6 months after the pandemic's onset. The comparison revealed a significant increase in fatigue levels (*P* < .001). Prepandemic, the median fatigue score was 4 (range, 0-8). After the onset of the pandemic, the score rose to 6 (range, 0-10) during the subsequent 6-months follow-up, confirming a significant role of the pandemic on fatigue in autoinflammation. PGA scores also increased during the pandemic period (*P* < .001). Disease activity and fatigue were significantly associated at all time points, but the increase in PGA scores was lower than that observed in fatigue scores (Fig 2).

### Factors associated with chronic fatigue

Among the 108 children and adults with CAPS, 83 (77%) met the definition for chronic fatigue. The distribution across different categories was as follows:

#### Gender

There was no significant association between gender and chronic fatigue (*P* = .87). Fatigue scores also did not significantly differ between genders (mean fatigue scores: females = 3.29, males = 3.90; mean difference = −0.62, *P* = .277, Cohen's *d* = −0.211).

#### NLRP3 variants

Thirty-six patients (43%) had pathogenic or likely pathogenic *NLRP3* variants, whereas 47 (57%) had a VUS or no mutation. The *NLRP3* variant was not associated with the status of chronic fatigue (*P* = .67). However, patients with pathogenic or likely pathogenic variants had a significantly higher mean fatigue score (4.44) compared to those with a VUS or no mutation (2.97), with a mean difference of −1.48 (*P* = .009) and an effect size of Cohen's *d* = −0.516.

#### Familial history

A family history of CAPS was documented in 51 of 83 patients (62%, *P* = .399). The mean fatigue score was higher in patients with a positive family history (3.94) compared to those without (3.07), but this difference was not statistically significant (mean difference = −0.87, *P* = .132, Cohen's *d* = −0.298).

#### Diagnosis age group

The majority of patients (53/83, 64%, *P* = .057) was diagnosed with CAPS in childhood. However, those diagnosed in adulthood had significantly higher mean fatigue scores (5.06) compared with childhood (2.91), with a mean difference of −2.15 (*P* < .001) and an effect size of Cohen's *d* = −0.776 (Table 2).

**Table 2:** Variables associated with chronic fatigue in CAPS

Category	Subgroup	N (%)	Mean fatigue score at last visit	Mean difference	*P* value^a^	Cohen's *d*
Gender	Female	40 (48)	3.29	−0.62	**.277**	−0.211
	Male	43 (52)	3.90			
*NLRP3* gene variant classification	Pathogenic/likely pathogenic	36 (43)	4.44	−1.48	**.009**	−0.516
	Variants of uncertain significance or none	47 (57)	2.97			
Family history of CAPS	Yes	51 (62)	3.94	−0.87	**.132**	−0.298
	No	32 (38)	3.07			
Age at diagnosis	Diagnosed in childhood	53 (64)	2.91	−2.15	**<.001**	−0.776
	Diagnosed in adulthood	30 (36)	5.06			

CAPS, cryopyrin-associated periodic syndromes.

*P* values <.05 indicate significance.

a Independent *t* tests were used to assess the association between variables and chronic fatigue.

### Risk model

In univariate analyses age group at diagnosis (childhood vs adulthood) and presence of a pathogenic or likely pathogenic *NLRP3* variant were significantly associated with the severity of chronic fatigue. When testing these variables in a multiple linear regression model, the model explained 13.9% of the variance in fatigue severity scores (*R*^2^ = 0.139), with a corrected *R*^2^ of 0.122, and was statistically significant overall (*F*[2, 105] = 8.463, *P* < .001). Age group at diagnosis was a significant negative predictor of fatigue severity (β = −0.292, *P* = .003), indicating that patients diagnosed in adulthood had significantly higher fatigue scores compared with those diagnosed in childhood.

## DISCUSSION

This study offers the first comprehensive evaluation of chronic fatigue in patients with CAPS, alongside a thorough assessment of disease activity. Fatigue was one of the most prevalent symptoms before treatment and has continued to be a significant concern even when the disease is well controlled. Despite successful treatment leading to control of disease activity and normalisation of inflammatory markers, fatigue scores remained elevated, underscoring that chronic fatigue persists and is not fully alleviated by the current disease management approaches.

Fatigue and disease activity were closely correlated, both decreasing in response to treatment. However, at the last visit, despite full disease control—evidenced by normalised clinical assessments, PGA scores, and laboratory inflammatory markers—fatigue levels remained elevated for many patients, significantly higher than the expected rates in the general population [16]. This indicates that controlling fatigue may require an additional management approach beyond traditional disease activity control. In several CAPS studies focused on canakinumab, a sustained response was observed in most patients, even after a single dose. This response was evident through both clinical symptom improvement and reduced levels of inflammatory markers. The role of IL-1 in disease control is an indisputable fact [5,6,[19], [20], [21]]. Studies by Koné-Paut et al [5] and Marsaud et al [6] investigated the effectiveness of canakinumab in treating CAPS-related fatigue. Similarly, improvements in CAPS-associated fatigue were observed in studies where rilonacept was used. Few studies have yielded comparable results when fatigue was assessed in paediatric populations after the administration of anakinra and canakinumab [[20], [21], [22], [23]]. However, to date, no comprehensive study has been conducted focusing on chronic fatigue in CAPS or other autoinflammatory diseases. Although IL-1β has traditionally been implicated in CAPS-related fatigue, the persistence of chronic fatigue under treatment suggests that other mechanisms may be involved [4,[24], [25], [26]]. Another hypothesis posits that high cortisol levels exert negative feedback on IL-1 levels. Chronic fatigue may be associated with dysfunction of the hypothalamic-pituitary-adrenal axis, eventually leading to hypocortisolism. This dysfunction could result in persistently high IL-1 levels due to the loss of diurnal variation in cortisol levels or during periods of stress [27,28]. The underlying mechanisms of fatigue remain unclear, necessitating further basic and translational research to elucidate these processes.

Individual factors play a significant role in chronic fatigue. Pathogenic or likely pathogenic *NLRP3* variants were associated with higher levels of chronic fatigue. Age at diagnosis also plays a crucial role; patients diagnosed in adulthood reported higher levels of fatigue compared with those diagnosed in childhood. Additionally, individuals with a family history of CAPS tended to report more chronic fatigue. Published studies examined individual factors and their influence on disease severity and sequelae, with similar findings showing that pathogenic mutations or severe phenotypes are associated with a more severe disease course or a greater need for higher treatment doses [[29], [30], [31]]. The prognosis for patients with CAPS is significantly influenced by early diagnosis, which facilitates the timely initiation of effective treatment. In untreated patients with CAPS, the prevalence of AA amyloidosis varies from 10% in mild phenotypes to 25% in moderate phenotypes. Additionally, early and aggressive treatment is essential for enhancing quality of life [1,32]. Moreover, the presence of a family history may indicate the likelihood of genetic factors and more severe mutations. Alongside these considerations, the phenomenon of learned behavioural fatigue must be acknowledged. The literature highlights that parental emotions, behaviours, and overall health play a critical role in shaping children's pain experiences [33]. However, there remains a notable gap in the literature regarding the relationship between individual factors and chronic fatigue in the context of autoinflammatory diseases. To our knowledge, this is among the first studies to address this issue, our research is particularly valuable and emphasises the necessity for personalised treatment objectives.

Our observations indicate a significant increase in fatigue scores during the COVID-19 pandemic in our CAPS cohort, reflecting a substantial impact on patients’ fatigue levels. Notably, a parallel increase in PGA scores was also observed during this period. This increase in disease activity could be attributed to challenges in accessing medication and medical care during the pandemic, as well as the presence of additional comorbidities. However, it is noteworthy that the rise in PGA scores was not as pronounced as the increase in fatigue scores. Therefore, it remains unclear to what extent this increase is driven by autoinflammatory disease itself or by pandemic-related individual and societal factors, as similar data are not available for other disease cohorts.

Overall, literature identifies fatigue as a notable feature of both acute and postacute COVID-19, with studies showing persistent fatigue in 13% to 33% of patients up to 20 weeks after symptom onset. This chronic fatigue has adversely affected patients’ quality of life, resulting in ongoing productivity losses and economic burdens [34]. Notably, 40 patients experience an average of 5.3 days of absenteeism every 3 months, despite the absence of active disease. The broader implications of the pandemic are also significant; prolonged fatigue has affected approximately 65 million individuals, resulting in an estimated societal cost of $2.6 trillion USD [35,36]. However, there is currently limited evidence on this issue, highlighting the need for more detailed research to understand the underlying mechanisms and long-term effects of fatigue in this context.

This study has several limitations. It is a single-centre observational study with a limited sample size, no control group and missing data. The study did not include assessments of patients’ quality of life, depression, or account for socioeconomic factors, which may have influenced the observed outcomes, nor did it include a healthy or other disease control group for comparison. However, the inclusion of a control group was not feasible due to the retrospective data analysis of the study. Nevertheless, prior research utilising the same fatigue VAS scoring method in patients with FMF reported a median fatigue score of 2 (IQR = 1) in healthy individuals [37]. In our cohort, the lowest recorded median fatigue score throughout longitudinal follow-up was 3 (IQR = 6), suggesting a consistently higher fatigue burden compared with the general population. Despite these limitations, the study uses validated instruments for disease activity assessment. The electronic patient record system ensures data consistency, maintaining quality despite the data gaps inherent to retrospective studies. Considering the exceptionally rare occurrence of CAPS, the study permits a meaningful analysis, yielding statistically significant results.

In conclusion, our findings emphasise the critical need for targeted and personalised therapeutic approaches to address chronic fatigue associated with autoinflammatory diseases, thereby improving patient outcomes and reducing the broader societal impact. Additionally, further research is required to explore underlying mechanisms and gather more evidence to inform and refine these therapeutic strategies.

## Competing interests

JBK-D has received research grants and speaker's fees from Novartis and SOBI. A-KG has also received speaker's fees from Novartis. The other authors declare no conflicts of interest.

## Appendix Supplementary materials

Image, application 1

## Footnotes

Supplementary material associated with this article can be found in the online version at doi:10.1016/j.ero.2025.03.003.
