# Supplementary material for: Burden of fatigue in cryopyrin-associated periodic syndromes
Source: EULAR Rheumatol Open. 2025 Apr 28;2(2):100006. doi: 10.1016/j.ero.2025.03.003 (PMC13425218; doi:10.1016/j.ero.2025.03.003)
Supplement: Supplementary file 1 [file mmc1.docx]

**Supplementary Table 1. Association Between Fatigue and Disease Activity (PGA) Over Time**

| **Timepoint** | **Median Fatigue Score (Range)** | **Median PGA Score (Range)** | **p-value** |
| --- | --- | --- | --- |
| **Baseline** | 7 (0–10) | 5 (0–9) | **<0.001** |
| **6 months** | 3.7 (0–9) | 0.8 (0–5) | **<0.001** |
| **12 months** | 3.3 (0–10) | 0.5 (0–6) | **<0.001** |
| **Last follow-up** | 3 (0–10) | 0.57 (0–8) | **0.013** |
